# The Protein Structure Context of PolyQ Regions

**DOI:** 10.1371/journal.pone.0170801

**Published:** 2017-01-26

**Authors:** Franziska Totzeck, Miguel A. Andrade-Navarro, Pablo Mier

**Affiliations:** 1 Faculty of Biology, Johannes Gutenberg University Mainz, Gresemundweg 2, Mainz, Germany; 2 Institute of Molecular Biology, Ackermannweg 4, Mainz, Germany; University of Pittsburgh School of Medicine, UNITED STATES

## Abstract

Proteins containing glutamine repeats (polyQ) are known to be structurally unstable. Abnormal expansion of polyQ in some proteins exceeding a certain threshold leads to neurodegenerative disease, a symptom of which are protein aggregates. This has led to extensive research of the structure of polyQ stretches. However, the accumulation of contradictory results suggests that protein context might be of importance. Here we aimed to evaluate the structural context of polyQ regions in proteins by analysing the secondary structure of polyQ proteins and their homologs. The results revealed that the secondary structure in polyQ vicinity is predominantly random coil or helix. Importantly, the regions surrounding the polyQ are often not solved in 3D structures. In the few cases where the point of insertion of the polyQ was mapped to a full protein, we observed that these are always located in the surface of the protein. The findings support the hypothesis that polyQ might serve to extend coiled coils at their C-terminus in highly disordered regions involved in protein-protein interactions.

## Introduction

Homopeptide repeats are consecutive stretches of the same amino acid in protein sequences. They are surprisingly common in proteins, and it has been suggested that they form unstructured stretches within a protein and may serve a function in protein-protein interaction (PPI) [1, 2]. Polyglutamine (polyQ) in particular is one of the most common homopeptide repeats in eukaryotic proteomes [1, 3]. It can be found in a variety of protein families which do not appear to be related [4].

Although there is an increasing amount of studies indicating the function of normal polyQ [5–8], most works study the effects of its abnormal extension, caused in human sequences by CAG trinucleotide expansion. There are several diseases known to be caused by such expansion of polyQ; most infamously Huntington’s disease [9]. All polyQ diseases involve neural degeneration, the onset and severity of which depends on the length of the expanded polyQ. Wild type huntingtin, for example, has a stretch of 23 glutamines; its expansion to less than 34 glutamines is not pathogenic, while a stretch of more than 36 glutamines results in neural degeneration [10, 11]. PolyQ proteins are known to be involved in PPIs [5], which may lead to aggregation when the polyQ is abnormally expanded. Indeed, polyQ diseases are characterised by aggregates of the respective protein containing an expanded polyQ tract within neuronal cells; however, their specific role in the disease is not quite clear. While these aggregates, as found in inclusion bodies, were originally thought to be the cause of polyQ diseases, later research suggested that they might serve a protective role and merely be a symptom of the disease rather than the cause [12].

Determination of the structure of polyQ regions has proven to be extremely difficult as they appeared to be very unstable. Most experimental studies seem to suggest a predominant random coil conformation [13–15]; however, there is also evidence for β-sheet as well as helical structure [16, 17]. Regarding the structure of aggregates, the current consensus is that these contain a high amount of β-sheets, possibly formed by a gradual change of conformation of polyQ proteins [18, 19]. While small polyQ-containing peptides have also been found to aggregate, it appears that expanded polyQ aggregates far more rapidly [13]. A subsequent recruitment of proteins with small polyQ tracts has also been reported [14].

Regarding the structure of the polyQ itself there is no consensus. It has been proposed that the structure of polyQ of both pathogenic and non-pathogenic length is largely the same [13], while some studies found a slight change in the overall secondary structure content [20], and others even reported a sharp change from an extended monomeric conformation to a collapsed state [21].

Recent studies have addressed the possible effect of the protein context on polyQ. It has been noted that many proteins containing polyQ also contain coiled coils, which facilitate protein interaction and, in case of polyQ expansion, participate in aggregation [22]. It was noted that polyQ appears often at the C-terminus of coiled coil regions and this was taken as an indication that polyQ could serve to increase the length of the coiled coil to modulate their interactions [5, 22]. We also noted that proteins that interact with polyQ proteins often contain coiled coils too and later we found evidence that their interaction with an expanded polyQ construct promotes its aggregation [23].

Other studies have addressed the possible effect of adjacent homorepeats on polyQ regions. polyP regions, which can often be found in the vicinity (C-terminal) of polyQ [5], were found to suppress aggregation of peptides containing a pathogenic polyQ stretch [24]. On the contrary, polyA regions can trigger polyQ aggregation through coiled coil formation [25].

Finally, additional studies concentrated on the domain context and provided evidence that indeed surrounding domains appear to play a role in polyQ aggregation [26–31]. However, an evaluation of the structural context of polyQ is as yet missing, although it is quite conceivable that secondary structure in the neighbourhood of polyQ might put constraints on its conformation.

This work focuses on the analysis of the natural protein structure context of polyQ, using the structures that have been determined so far of both polyQ proteins and their homologs for an analysis of the secondary structure around it.

## Methods

### Definition of polyQ

PolyQ was defined as a consecutive stretch of amino acids that contains at least eight glutamines per ten residues (a maximum of two mismatches in a minimum of ten residues); however, other definitions for polyQ were also tested to check the dependence of the results. PolyQ thresholds are written as a fraction, where the denominator indicates the number of amino acids and the numerator the minimum number of glutamines found in it (e.g. 8/10 means a threshold of at least eight glutamines per ten amino acids).

### Dataset

The dataset was obtained using FastaHerder2 mode 4 [32] to look for clusters of similar protein sequences of comparable length containing at least one protein with polyQ (in FastaHerder2 defined as 8/10) and at least one protein with associated PDB annotation. Clusters were generated from SwissProt proteins (548,454 proteins, release 2015_05), and the proteins within them are at least 53% identical. Theoretical protein structures were not taken into account, and clusters for which only theoretical models were available were dismissed. This produced a total of 178 clusters, 74 of which consisted only of one protein. All in all the dataset contained 926 proteins.

Protein sequences were downloaded from the UniProt database (http://www.uniprot.org) [33]. Clusters containing multiple proteins were aligned using Clustal Omega [34] on the UniProt web server [33] with default parameters. All structure files were obtained from the RCSB PDB (http://www.rcsb.org/pdb/home/home.do) [35]. Pictures of protein structures were produced using UCSF Chimera 1.10.2 [36].

### Script for analysis

The script used to analyse the data was written in Java programming language. Secondary structure information was extracted from the PDB files with the aid of BALL 1.4 [37], for which a small C++ script was written and called from the main Java script. The analysed data, the main Java script and the helper script in C++ can be found in our web server (http://cbdm-01.zdv.uni-mainz.de/~munoz/polyq/).

### Obtaining secondary structure information for a protein

Information extracted from the PDB files was gathered for every residue of every chain by retrieving its index number, amino acid type and secondary structure. The secondary structure contained the information whether a given residue of the chain had the geometric properties of a residue within a helix, sheet or random coil. Subsequently, a matching of the amino acid sequence of the chain and the respective protein was attempted; at first by using the index. However, many PDB structures contain information from several proteins, making it necessary to compare also the sequence identity of both chain and respective part of the protein. Furthermore, many proteins are in multiple PDB files, which often contain multiple chains of the same sequence. In order to obtain one coherent structure string, for every position either the secondary structure with the best resolution was used, or, if resolutions were the same but structures differed, an identical likelihood for both conformations was assumed for that position.

Most polyQ proteins in the dataset have no associated structure information. However, protein structure is more conserved than protein sequence. The structure of related proteins is very likely to be similar to that of the protein of interest itself. For some polyQ proteins, more than one of the related proteins had structure information for a given position. If this was the case, all of the possible conformations at that position were taken into account and normalised for the total amount of secondary structure information at that particular position (e.g. if two proteins had a helical conformation and one a random coil, the structure was counted as 0.66 helical and 0.33 random coil). If the polyQ protein happened to have own secondary structure information for a given position, none of the structures of related proteins were taken into account.

### Normalisation of the data

There was a varying amount of polyQ proteins per cluster; furthermore, some proteins contained more than one polyQ. In this analysis, the data were normalised for the number of polyQ, regardless of the number of actual proteins. For example, if a cluster had two polyQ proteins, one with one polyQ and the other with four polyQs, each of these polyQs was taken into account individually and added up; subsequently, the sum for each position of the secondary structure information (helix, sheet, random coil or no information) was divided by five.

## Results

A set of 178 clusters, consisting of polyQ proteins and their homologs (at least a 53% identity between proteins in a cluster, see Methods for details), was analysed for secondary structure context in the vicinity of polyQ regions. Homologs were taken into account in order to increase the amount of secondary structure information, which was categorised into helix, sheet or random coil. The clusters in the dataset were of varying size, from one to 243 proteins. A total of 282 proteins out of the 926 in the dataset contain a polyQ with at least eight glutamines per ten amino acids (an 8/10 polyQ). Most of them only contain one polyQ, but there are a few that contain a higher number, up to six.

### The protein structure context of polyQ

**Fig 1:**
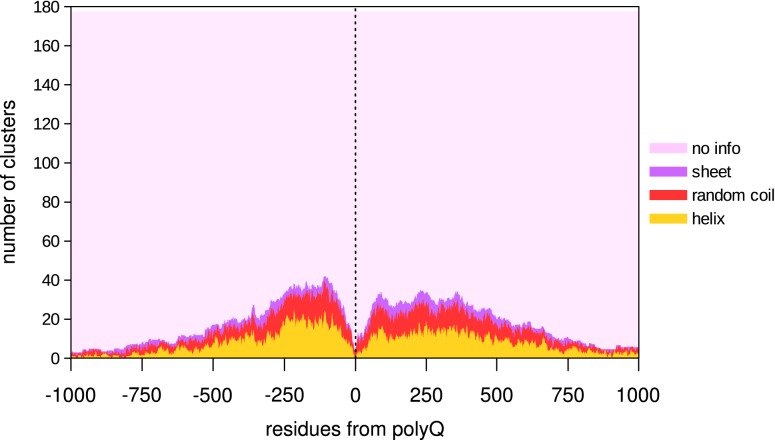
Structural information per residue surrounding the polyQ stretch (8/10). Each residue could either have no structural information (“no info”, pink), or have the information: sheet (purple), random coil (red) or helix (yellow). The drop of secondary structure information at the sides of the graph only reflects the size distribution of the fragments used.

Generally, structure information is available only for fragments of proteins and not for complete proteins, firstly because usually only parts of proteins are studied, but also because some regions might be disordered and will not adopt a single structure that could be resolved. The latter effect greatly influenced our observations since for most of the clusters we used there was no structural information available in the closer proximity of the polyQ (Fig 1).

Despite the fact that all clusters have some structural information, a feature for which they were selected, only a maximum of around 40 clusters have overlapping information for any given residue in relation to the polyQ position. In any case, taking into account the structure's results, these show a prevalence of helical structure as opposed to random coils and β-sheet (range -500:500).

**Fig 2:**
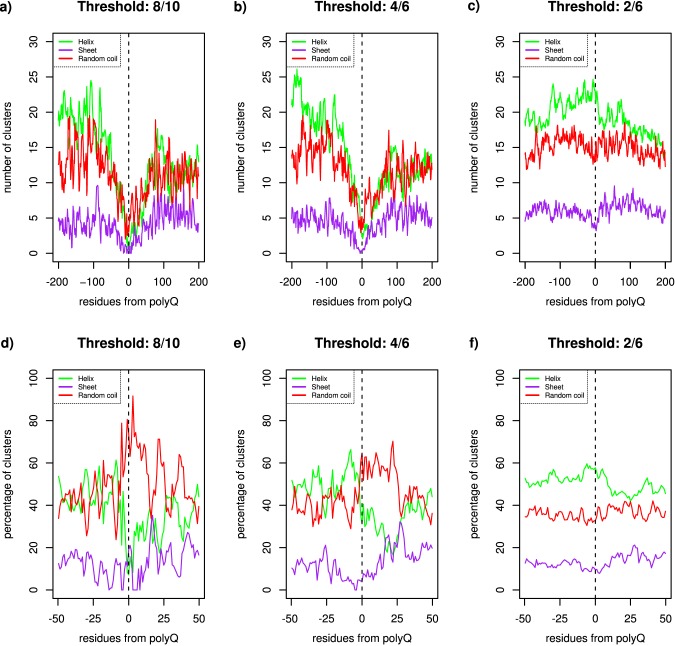
Structure context of polyQ regions using different thresholds. Number (a, b, c) or percentage (d, e, f) of clusters with a certain structural conformation per residue surrounding the polyQ stretch, using a 8/10 (a and d), 4/6 (b and e), or 2/6 (c and f) polyQ threshold.

Next, we analysed the distribution of structures for various thresholds for polyQ definition (Fig 2). In the -200:200 range of the polyQ, there is an overall prevalence of both helix and random coil conformation; sheet conformation is particularly rare close and N-terminal to the polyQ (Fig 2A). Considering the immediate proximity of the polyQ (range -25:25), the N-terminal region is more likely to be in helical structure than the C-terminal region. This can be better seen when the data are normalised by the number of clusters with structural information for each position: while the ratio of helical structure is roughly between 40% and 60% in the closest positions to the polyQ origin (range -25:5), it is clearly lower in the closest C-terminal positions (range 5:25), roughly between 20% and 40% (Fig 2D). The results suggest that helical structures are preferably N-terminal to the polyQ middle position, while random coils are preferably C-terminal to it. Since position 0 is taken as the middle of the polyQ, the distribution of structures also shows that the secondary structure most likely to overlap with the middle of a polyQ is a random coil or a helix, if there is a solved structure for the region at all. In this respect, a very strong observation is the lack of solved structure close to the 0 (Fig 2A and 2B).

Results may differ depending on the threshold used to consider a polyQ. To characterise in detail the impact a polyQ region may have on the protein structure, several different polyQ definitions were also used. Using a 6/8 polyQ, the amount of available structural data increases slightly, while the ratio of structures in the vicinity of the polyQ still looks largely the same as that for 8/10 (data not shown). This suggests that a 6/8 polyQ is behaving the same way in relation to the rest of the protein and its structural context as an 8/10 polyQ.

A similar result is achieved when the definition of polyQ is lowered down to 4/6 (Fig 2B and 2E). The absence of sheet conformation close and N-terminal to the polyQ remains, but in the longer range it becomes more apparent that there is more sheet content in the 0:200 than in the -200:0 range, a bias that we cannot explain. Regarding the distribution of helix and random coil in the -25:25 range of polyQ, now, almost 40% of the structure overlapping with polyQ is a helix (Fig 2E). The valley of low helix conformation near polyQ is still present but shifted about 25 residues towards the C-terminus of the polyQ. The polyQ structural context differs considerably when using a definition of polyQ as weak as 2/6. The secondary structures are almost evenly distributed around the polyQ, independently of the distance to it (Fig 2F). The available structural information is not reduced in the proximity of the polyQ region (no valley close to position 0) (Fig 2C), which suggests that a 2/6 threshold should not be considered as a polyQ, as the structure around it is not disrupted by its presence.

### Places of polyQ insertion in experimental protein structures

**Fig 3:**
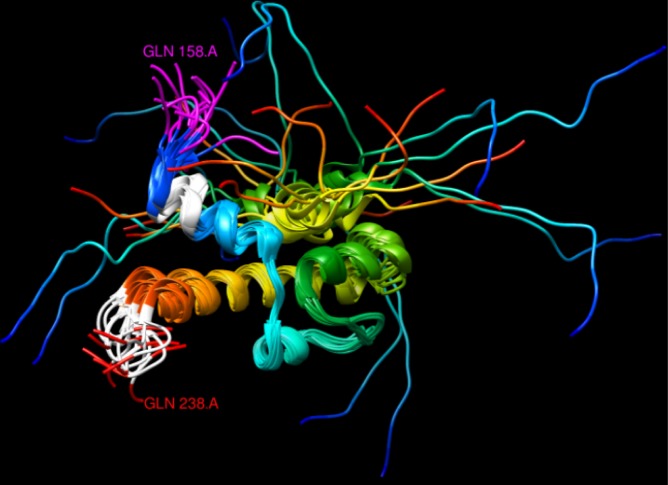
NMR structure of yeast protein Gal11 (PDB:2LPB), residues 158–238. One 4/4 and another 6/8 polyQ regions are coloured in white (top and bottom, respectively), while the last amino acids of a 12/12 polyQ are coloured in pink. The protein structure is shown with a ribbon representation and non polyQ regions were coloured from blue to red according to the sequence position from N- to C-terminal, respectively. The overlapping structures represent an ensemble of models from the nuclear magnetic resonance (NMR) spectra of the protein in solution.

While, as shown above, polyQ regions are generally not part of solved structures, there were a few cases where the structure of a polyQ region was available. Among glutamine stretches associated with helices, both N- and C-terminal locations could be found. In the mouse protein OTX2 (UniProt:P80206), for example, a 7/7 polyQ is at the C-terminus of a helix (PDB:2DMS), while in the human CREB-binding protein (UniProt:Q92793) a 5/5 polyQ is present at the N-terminus of a helix (PDB:1ZOQ). Interestingly, in the structure of the yeast protein Gal11 (UniProt:P19659, PDB:2LPB) both cases happen, as one 4/4 polyQ is present N-terminal to a helix and another 6/8 polyQ is C-terminal to another helix (Fig 3, in white).

Although the majority of structures in the dataset were from protein fragments, a few of them were nearly complete proteins, like the structures of the proteins WHY1 from *Solanum tuberosum* (PDB:1L3A), WDR5 from *Rattus norvegicus* (PDB:4QQE), glycinin G1 from the soybean (PDB:1FXZ) and SEC23 from *Saccharomyces cerevisiae* (PDB:1M2O). In all four of these structures, the part of the structure closest to the polyQ region (or its place of insertion, when considering homologous proteins) is located at the outside of the protein (data not shown). Such exposed position of the polyQ in relation to the global structure of a protein supports its involvement in protein-protein interactions (PPIs), as polyQ needs to be placed on the outside of the folded protein to have a role in PPI.

## Discussion

We previously reported the co-occurrence of coiled coils and polyQ by sequence analysis of proteins containing polyQ [5]. There, these findings were complemented with the analysis of the protein interaction networks surrounding polyQ proteins (e.g. finding that polyQ proteins tend to interact with other polyQ proteins). Here, we used a different set of sequences, which for the most part consists of proteins of known 3D structure without polyQ but homologous to polyQ proteins. This allowed us to have a focus on the study of the structure surrounding polyQ while at the same time avoiding the problem of lack of 3D structures of polyQ regions.

We found that polyQ is generally located in a helix / random coil context where the helix is preferably N-terminal and the random coil preferably C-terminal to the polyQ middle position. This pattern of secondary structure distribution in relation to the polyQ position was also detected for lower thresholds of polyQ, like 4/6. This suggests that polyQ function is linked to a helix / random coil structure context, and that even short stretches of repeats can serve this function.

The few examples of polyQ stretches in experimental structures are all located at helix terminals. However, these could be found C- as well as N-terminal to a helix. Possibly the function of polyQ is improved if it is positioned towards the C-terminus of a helix but still takes place even at the N-terminus.

Lately, it was proposed that polyQ might extend coiled coils. A recent study found that polyQ proteins often contain coiled coils and that aggregation of polyQ proteins can be promoted by enhancing coiled coil propensity. The authors suggested that the polyQ might enhance protein-protein interaction through this coiled coil extension [22]. The finding of co-occurrence of polyQ and coiled coils was later supported by our own work [5]. Here, relying on protein sequences of known structure most often lacking the polyQ but homologous to the region surrounding a polyQ in another protein, we have shown that the point of insertion of polyQ is N-terminal to a region enriched in alpha-helix. While theoretical coiled-coil predictions might be unreliable for polyQ proteins, collectively previous work seems to suggest a relation between polyQ and coiled-coils; here, we suggest that polyQ is often inserted right after regions with alpha-helical structure, and that, in our opinion, would be consistent with their role in extending such type of structure. We expect that experimentally verified 3D structures will eventually confirm this association. For example, a helical polyQ structure is found at the C-terminus of a coiled coil in the solved structure of the N-terminus of huntingtin [38].

Interestingly, in every structure we found where polyQ was included, the polyQ was located towards the outside of the protein, which further supports a general function for polyQ in protein-protein interaction.

PolyQ proteins are known to be often unstructured and as such difficult to crystallize [16]. Our results indicate that this is not only due to polyQ itself, but also possibly due to the place where polyQ is inserted in evolution since the lack of solved structures affects not just the polyQ protein but also the homologs of polyQ proteins that lack the polyQ (Fig 1; S1 Fig). Interestingly, the structural information reaches levels similar to the background when using a 2/6 threshold for polyQ, which suggests that indeed it should not be considered as a polyQ region.

Though the findings of our study are compelling, the scarcity of the data rather impacts their reliability. While there seems to be a clear tendency for a helix / random coil context surrounding polyQ, one should use the present findings to investigate further. In particular the suggestion that lower thresholds of polyQ definition show a similar structure context as higher ones might help defining functional polyQ regions, which should contribute to our understanding of the functions and interactions of many proteins.

## Supporting Information

S1 Fig Structural information per residue surrounding the place where the polyQ (8/10) is inserted in evolution, only taking into account proteins without polyQ. Each residue could either have no structural information (“no info”, pink), or have the information: sheet (purple), random coil (red) or helix (yellow). The drop of secondary structure information at the sides of the graph only reflects the size distribution of the fragments used.
(PDF)
