# Supplementary figures and images for: The Protein Structure Context of PolyQ Regions
Source: PLoS One. 2017 Jan 26;12(1):e0170801. doi: 10.1371/journal.pone.0170801 (PMC5268486; doi:10.1371/journal.pone.0170801)

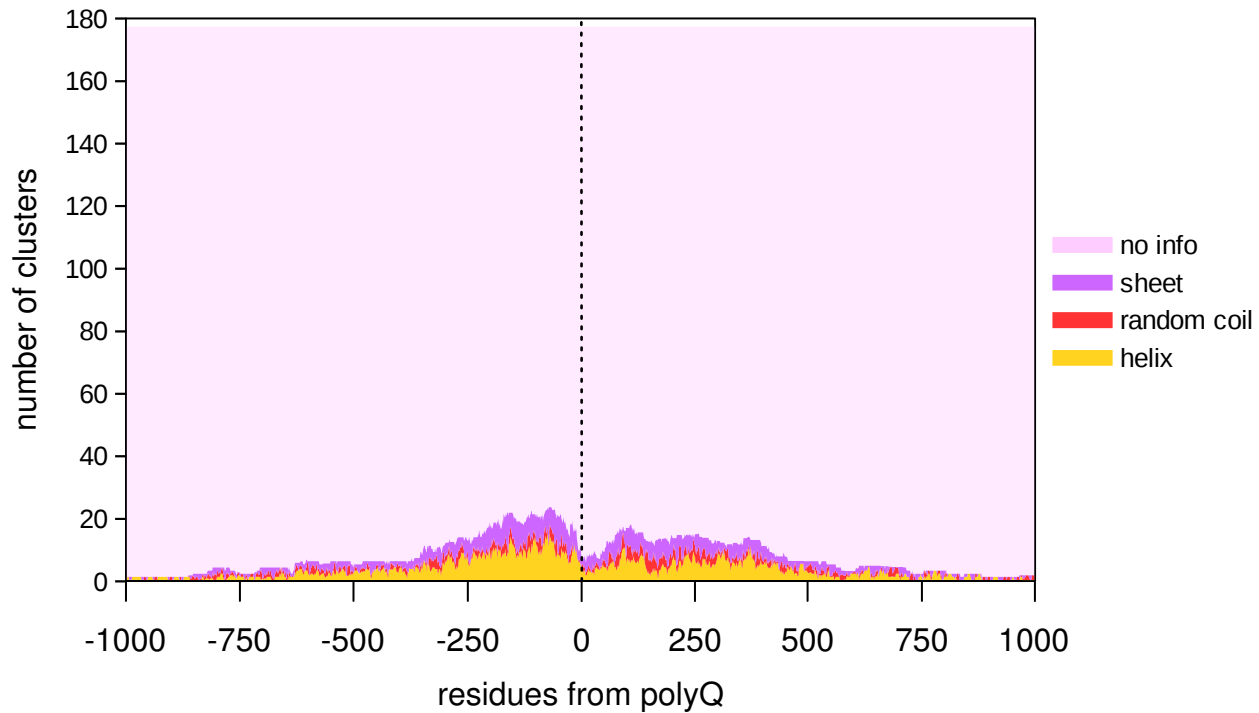

Supplement: S1 Fig — Each residue could either have no structural information (“no info”, pink), or have the information: sheet (purple), random coil (red) or helix (yellow). The drop of secondary structure information at the sides of the graph only reflects the size distribution of the fragments used. (PDF) [file pone.0170801.s001.pdf]
